# Potential of ciprofol as an alternative to propofol in elderly patients undergoing gastrointestinal endoscopy: a meta-analysis and trial sequential analysis

**DOI:** 10.3389/fphar.2025.1644182

**Published:** 2025-08-11

**Authors:** Yunfeng Yu, Min Liao, Juan Deng, Xinyu Yang, Yuman Yin, Zhenjie Liu, Guomin Zhang

**Affiliations:** ^1^ School of Integrated Chinese and Western Medicine, Hunan University of Chinese Medicine, Changsha, Hunan, China; ^2^ Department of Anesthesiology, People's Hospital of Ningxiang City, Changsha, Hunan, China; ^3^ Department of Gastroenterology, The First Hospital of Hunan University of Chinese Medicine, Changsha, Hunan, China

**Keywords:** ciprofol, propofol, elderly patients, gastrointestinal endoscopy, meta-analysis

## Abstract

**Objective:**

Ciprofol is increasingly being used for sedation and induction of anesthesia in China. However, it remains unclear whether ciprofol is a more appropriate sedative than propofol in gastrointestinal endoscopy, especially in the elderly population. This study aimed to compare the safety of ciprofol with propofol in elderly patients undergoing gastrointestinal endoscopy.

**Methods:**

Eight common databases were used to search the relevant literature up to 1 January 2025. Included studies were screened according to established criteria, and their basic characteristics, outcome data, and risk of bias were recorded. Subsequently, Review Manager 5.3 software was used to perform meta-analysis and Trial Sequential Analysis (TSA) 0.9.5.10 Beta software was used to perform TSA.

**Results:**

Twelve randomized controlled trials and 1,653 participants were included in this study. Meta-analysis showed that compared to propofol, ciprofol reduced the incidence of hypotension (risk ratio [RR] 0.59, 95% confidence interval [CI] 0.48–0.71, *P* < 0.00001), respiratory depression (RR 0.30, 95% CI 0.20–0.46, *P* < 0.00001), hypoxemia (RR 0.29, 95% CI 0.20–0.43, *P* < 0.00001), injection pain (RR 0.15, 95% CI 0.10–0.22, *P* < 0.00001), involuntary movements (RR 0.70, 95% CI 0.53–0.92, *P* = 0.01) as well as nausea and vomiting (RR 0.59, 95% CI 0.36–0.96, *P* = 0.03), while there was no significant effect on induction time, awakening time, bradycardia, and choking cough (*P* > 0.05). The TSA revealed conclusive differences in hypotension, respiratory depression, hypoxemia, and injection pain. Regression analysis indicated no publication bias for any of the outcomes (*P* > 0.05) except awakening time (*P* = 0.007).

**Conclusion:**

These findings suggest that in elderly patients undergoing gastrointestinal endoscopy, ciprofol has fewer cardiovascular, respiratory, and neurological adverse events than propofol, highlighting the potential of ciprofol as an alternative to propofol. However, the optimal dose of ciprofol for gastrointestinal endoscopic sedation in the elderly remains to be explored.

**Systematic Review Registration:**

https://www.crd.york.ac.uk/PROSPERO/view/CRD42025643465, identifier CRD42025643465.

## 1 Introduction

Population aging has become an irreversible global trend as the number and proportion of elderly people in various countries continue to rise (Partridge et al., 2018). According to the World Health Organization, the population aged 60 years or older is projected to increase from 1 billion in 2020 to 1.4 billion in 2030 and grow to 2.1 billion in 2050 (Aging and Health, 2025). With the intensification of global aging, the medical needs of the elderly are experiencing a rapid surge (Christensen et al., 2009). 85% of the elderly are reported to suffer from one or more chronic diseases, especially gastrointestinal diseases (Cristina and d’Alba, 2021). Gastrointestinal endoscopy is the gold standard for diagnosing digestive disorders and plays a crucial role in the screening of gastrointestinal disorders such as chronic atrophic gastritis, esophageal cancer, gastric cancer, and colorectal cancer, which are prevalent in the elderly (Finocchiaro et al., 2021). Nevertheless, gastrointestinal endoscopy is an invasive procedure that can lead to adverse events such as pain, nausea, vomiting, choking, and anxiety suffered by the patients examined (Smale et al., 2003). These not only cause pain and discomfort to the patient, but also reduces the accuracy and speed of the examination (Mitsonis et al., 2011).

Therefore, the guidelines for sedation and anesthesia for gastrointestinal endoscopy recommend the use of sedative in gastrointestinal endoscopy to reduce the discomfort of the patient and improve the success rate of the examination (Early et al., 2018). With the gradual popularization of procedural sedation in gastrointestinal endoscopy, both the acceptance and experience of the operation among patients have steadily improved (Sidhu et al., 2024; Benzoni and Cascella, 2025). As a classic short-acting intravenous sedative, propofol has long been recognized as the preferred sedative for gastrointestinal endoscopy (Ferreira and Cravo, 2015). However, propofol induces side effects of cardiovascular and respiratory depression when depressing the central nervous system, which is particularly prevalent in the elderly population (Short and Bufalari, 1999). Reports indicate that the incidence of hypotension, respiratory suppression, and hypoxemia in elderly patients undergoing gastrointestinal endoscopy with propofol is 69.6%, 10.0%, and 17.4%, respectively (Lu et al., 2022; Liu et al., 2023). Therefore, the potential risks of cardiovascular and respiratory depression associated with propofol in elderly patients undergoing gastrointestinal endoscopy are a cause for concern. This has prompted anesthesiologists to continually seek safer sedation strategies for this vulnerable patient group.

**FIGURE 1:**
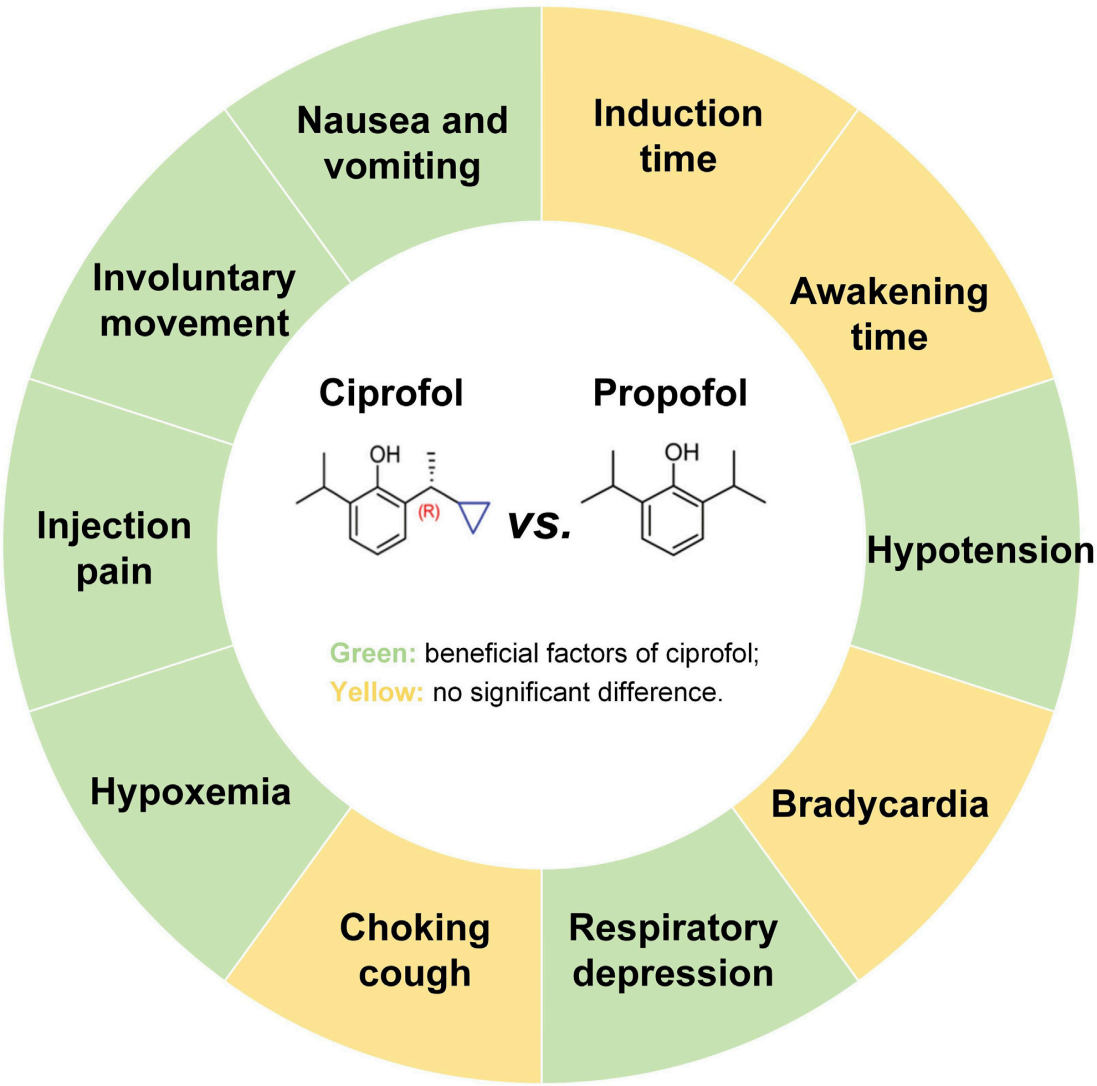
Graphical abstract.

Ciprofol is a new type of short-acting intravenous anesthetic independently developed in China, which is a derivative of propofol. The mechanisms of action of ciprofol and propofol are similar, and both are characterized by rapid onset of action and rapid metabolism. However, the inhibitory effects of ciprofol on the cardiovascular and respiratory systems are milder than those of propofol (Task Force on Guidelines on Clinical Application of Ciprofol, 2023; Lu et al., 2023). Ciprofol was first approved for gastrointestinal endoscopic sedation by the National Medical Products Administration in 2021 and is under review by the Food and Drug Administration (The National Medical Products Administration, 2020; Huanbo, 2024). A previous meta-analysis showed that ciprofol reduced adverse events such as hypotension, bradycardia, respiratory depression, and hypoxemia in general anesthesia in the elderly compared to propofol, highlighting the value of ciprofol in the elderly population (Chen et al., 2025). A subsequent clinical study demonstrated that patients sedated with ciprofol exhibited more stable hemodynamics compared to those sedated with propofol, suggesting that ciprofol has potential as an alternative to propofol for gastrointestinal endoscopy (He et al., 2024). However, no relevant meta-analysis has reported differences between ciprofol and propofol in procedural sedation for the elderly, especially in gastrointestinal endoscopic sedation. Therefore, in this study, we employed both meta-analysis and trial sequential analysis (TSA) to comprehensively compare the safety of ciprofol and propofol in sedation for elderly patients undergoing gastrointestinal endoscopy, aiming to provide evidence for the clinical use of ciprofol. The main findings of this study are shown in Figure 1.

## 2 Methods

This study strictly followed Preferred Reporting Items for Systematic reviews and Meta-Analyses (PRISMA) (Page et al., 2021) and was registered in PROSPERO (CRD42025643465).

### 2.1 Literature search

The articles were searched across English databases such as PubMed, Embase, the Cochrane Library, and Web of Science, as well as Chinese databases including China National Knowledge Infrastructure (CNKI), China Science and Technology Journal Database (CSTJ), WanFang, and SinoMed. The search field was set to Title/Abstract, and the search formula was set to ((Ciprofol OR Cyclopropanes) AND (Endoscopy OR Endoscope OR Endoscopies OR Endoscopic OR gastroscope OR gastroscopy OR colonoscope OR colonoscopy OR sigmoidoscopy)). The search was conducted up until 1 January 2025, with no restrictions on language or others.

### 2.2 Inclusion and exclusion criteria

Inclusion criteria: (1) Study design: randomized controlled trials (RCTs); (2) Participants: elderly individuals undergoing upper gastrointestinal endoscopy or lower gastrointestinal endoscopy, with no significant abnormalities in coagulation or liver function. The gastrointestinal endoscopy procedures were conducted according to established protocols, including comprehensive steps for cleaning, disinfection, rinsing, and drying/storing. (3) Intervention: the experimental group received ciprofol sedation; (4) Comparison: the control group received propofol sedation; (5) Outcomes: time-related outcomes included induction time and recovery time; cardiovascular outcomes included hypotension and bradycardia; respiratory outcomes included respiratory depression, choking cough, and hypoxemia; neurological outcomes included injection pain and involuntary movements; gastrointestinal outcomes included nausea and vomiting.

Exclusion criteria: (1) Repeatedly published studies; (2) Studies with missing baseline data; (3) Studies with unavailable data; (4) Studies involving endoscopic treatments such as endoscopic retrograde cholangiopancreatography.

### 2.3 Literature screening

Initially, all retrieved studies were imported into Zotero 7.0, where duplicates were automatically removed using the software. Subsequently, the titles, authors, journals, volumes, issues, and DOIs of the remaining studies were manually verified, and any remaining duplicates were eliminated. Subsequently, by reviewing the title and abstract, further studies unrelated to the research topic were excluded. Finally, the full texts of the remaining studies were examined to confirm the final selection. Every step of the literature screening was independently conducted and cross-verified by YY and JD, with any disagreements resolved by XY.

### 2.4 Data collection

Excel 2010 was utilized to create statistical tables recording the basic characteristics and outcome data for included studies. The basic characteristics table included the first author, publication year, participant source, sample size, male-to-female ratio, average age, body mass index (BMI), ASA classification, anesthesia induction, and operation type. Any data related to outcomes were recorded in the data statistics table. Each step was independently performed and cross-verified by YY and JD, with any disagreements resolved by XY.

### 2.5 Risk assessment of bias

The bias risk assessment tool provided by the Cochrane Collaboration was employed to evaluate the methodological quality of the included studies. The tool encompasses aspects such as random sequence generation, allocation concealment, blinding of participants and personnel, blinding of outcome assessment, incomplete outcome data, selective reporting and other biases. Each aspect was classified as low risk, unclear risk, or high risk. The bias risk assessment was independently carried out and cross-checked by YY and JD, with any disagreements settled by XY.

### 2.6 Statistical analysis

Review Manager 5.3 software was utilized to perform meta-analysis, subgroup and sensitivity analyses, as well as publication bias assessment. Firstly, the risk ratio (RR) was set as the effect size for dichotomous variable, whereas the mean difference (MD) was employed for continuous variables. If the measurement methods were identical, the weighted mean difference (WMD) was adopted; otherwise, the standardized mean difference (SMD) was used. The *I* ^2^ test was conducted to evaluate the heterogeneity across the included studies. An *I* ^2^ value of <50% indicated no significant heterogeneity, leading to the use of a fixed-effect model for the meta-analysis; when *I* ^2^ was ≥50%, indicating moderate to high heterogeneity, a random-effects model was applied. Secondly, subgroup and sensitivity analyses were conducted to explore and identify sources of heterogeneity. Subgroup analysis was performed based on factors such as male-to-female ratio, average age, BMI, ASA I ratio, and propofol dose. Sensitivity analysis, conducted using the leave-one-out method, aimed to identify sources of heterogeneity. Thirdly, Egger and Harbord regression analyses were employed to assess publication bias for continuous and dichotomous variables, respectively. Egger regression analysis is a method to evaluate publication bias by weighted regression of effect estimates and their precision. Harbord regression analysis is a technique to correct Egger linear regression using score test statistics and their variance, particularly suited for publication bias assessment of dichotomous variables. A *P* value of ≥0.1 in the regression analyses indicated no potential publication bias.

TSA 0.9.5.10 Beta software was used to perform sequential analysis. A type I error rate of 5% and a type II error rate of 20% were set, and MD, standard deviation, and relative risk reduction were calculated based on the included studies to conduct a bidirectional trial sequential analysis. When the cumulative Z curve crossed the trial sequential monitoring boundary, the current data were deemed sufficient to draw a definitive conclusion, and further data collection was unnecessary.

The “Grading of Recommendations, Assessments, Development and Evaluations” (GRADE) guideline was used to evaluate the certainty of evidence. This method classifies the certainty of evidence into high, moderate, low, and very low categories based on factors such as risk of bias, inconsistency, indirectness, imprecision, and publication bias.

## 3 Results

### 3.1 Literature screening process

**FIGURE 2:**
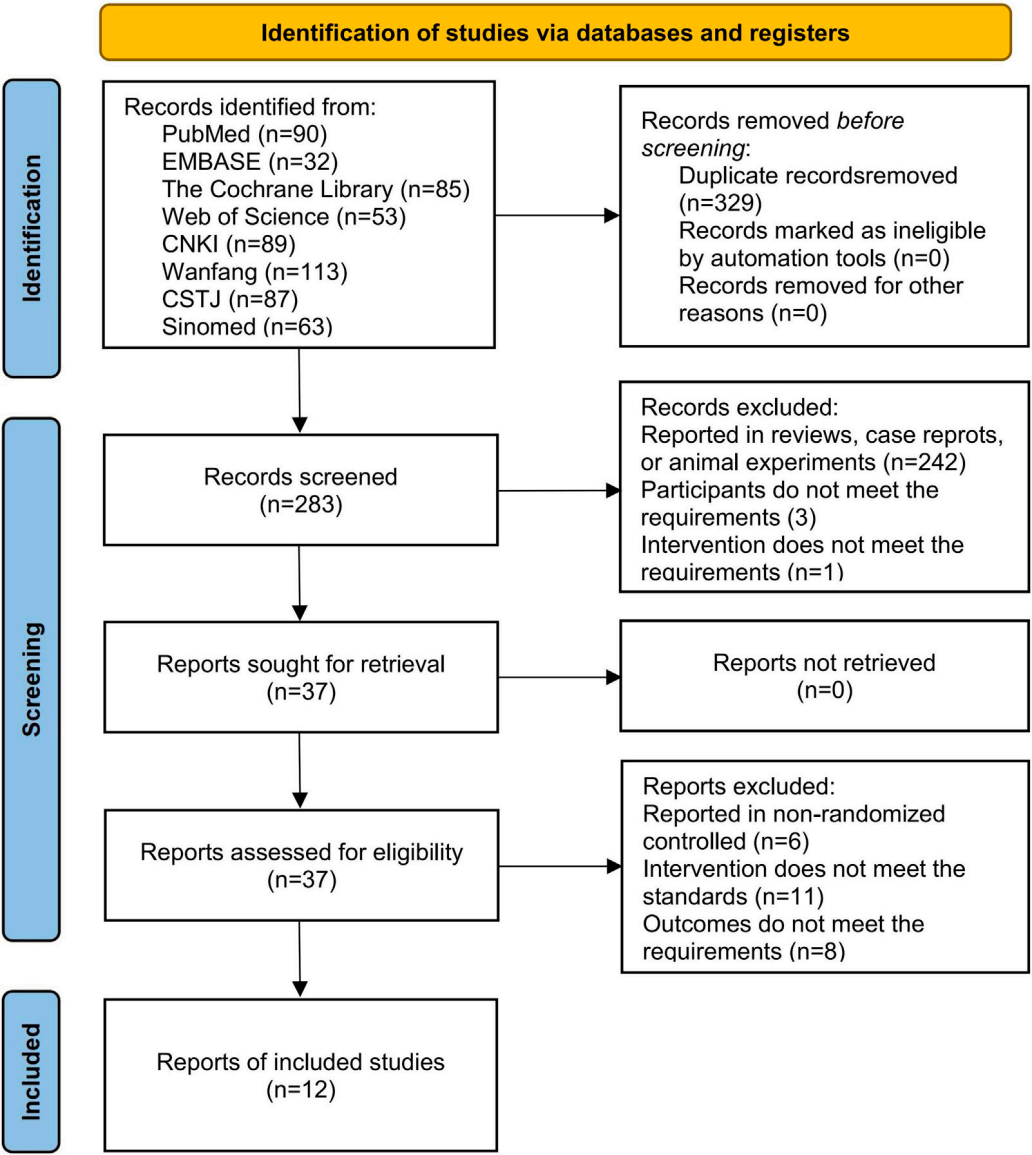
Flowchart of literature screening.

We obtained a total of 612 studies from eight databases, 90 from PubMed, 32 from EMBASE, 85 from the Cochrane Library, 53 from Web of Science, 89 from CNKI, 87 from CSTJ, 113 from Wanfang, and 63 from Sinomed. During the screening process, 329 studies were excluded due to duplicates, and 242 studies were excluded due to irrelevant topics. Subsequently, 29 studies were excluded due to not meeting the inclusion criteria when reviewing the full text. Among these, 6 studies reported non-RCT, 11 studies reported inconsistent interventions, and 8 studies reported unavailable outcomes. Finally, 12 studies (Gao et al., 2023; Huo and Dan, 2024; Liu Q. Z. et al., 2024; Ma and Huang, 2023; Wang et al., 2023; Xin, 2023; Xu et al., 2023; Yang et al., 2023; Yao et al., 2024; Yi et al., 2022; Yuan and Chen, 2024; Zhang and Zhu, 2023) were included in this analysis (Figure 2).

### 3.2 Basic characteristics of included studies

We included a total of 12 RCTs (Gao et al., 2023; Huo and Dan, 2024; Liu Q. Z. et al., 2024; Ma and Huang, 2023; Wang et al., 2023; Xin, 2023; Xu et al., 2023; Yang et al., 2023; Yao et al., 2024; Yi et al., 2022; Yuan and Chen, 2024; Zhang and Zhu, 2023) and 1,653 elderly individuals undergoing gastrointestinal endoscopy. Among them, 825 participants received ciprofol sedation and 828 participants received propofol sedation. The publication dates of the included studies were all in the last 3 years, the experimental centers were all in China, and the participants were all Chinese. The mean male ratio of the participants was 50.8%, the mean age was 69.70 years, the mean BMI was 24 kg/m^2^, the mean ASA I ratio was 32.05%, and the range of propofol anesthesia induction doses was 0.3–0.6 mg/kg (Table 1). In addition, none of the included studies reported comorbidity and their ratios.

**TABLE 1:** Basic characteristics of included studies.

Author name	Sample	Male/%	Age/years	BMI/(kg·m^−2^)	ASAI/%	Intervention	Comparison	Examination type
Gao et al. (2023)	61/60	40.5	67.7	24.6	24.8	Ciprofol 0.3–0.4 mg/kg	Propofol 1.2–1.6 mg/kg	Gastrointestinal endoscopy
Huo and Dan (2024)	82/82	53.7	69.6	23.8	38.4	Ciprofol 0.5–0.6 mg/kg	Propofol 1.5 mg/kg	Gastroscopy
Liu J. et al. (2024)	40/40	43.8	69.5	—	—	Ciprofol 0.4 mg/kg	Propofol 2.0 mg/kg	Gastrointestinal endoscopy
Ma and Huang (2023)	40/40	48.8	65.3	—	56.3	Ciprofol 0.3 mg/kg	Propofol 1.5 mg/kg	Gastroscopy
Wang et al. (2023)	49/50	54.6	76.4	24.3	54.5	Ciprofol 0.2 mg/kg	Propofol 1.0 mg/kg	Colonoscopy
Xin (2023)	40/40	43.8	70.2	23.1	30.0	Ciprofol 0.3 mg/kg	Propofol 1.5 mg/kg	Gastroscopy
Xu et al. (2023)	164/166	54.2	69.3	24.5	15.8	Ciprofol 0.4 mg/kg	Propofol 2.0 mg/kg	Colonoscopy
Yang et al. (2023)	30/30	58.3	68.1	24.5	26.7	Ciprofol 0.4 mg/kg	Propofol 1.0–2.0 mg/kg	Gastrointestinal endoscopy
Yao et al. (2024)	90/90	53.9	75.2	25.7	55.6	Ciprofol 0.3–0.4 mg/kg	Propofol 1.2–1.6 mg/kg	Gastrointestinal endoscopy
Yi et al. (2022)	79/80	52.2	69.9	23.7	12.5	Ciprofol 0.2 mg/kg	Propofol 1.0 mg/kg	Gastroscopy
Yuan and Chen (2024)	100/100	48.5	65.0	23.2	—	Ciprofol 0.3 mg/kg	Propofol 1.5 mg/kg	Gastrointestinal endoscopy
Zhang and Zhu (2023)	50/50	57.0	70.5	22.8	6.0	Ciprofol 0.3 mg/kg	Propofol 1.5 mg/kg	Gastroscopy

There were no significant differences in male ratio, age, BMI, and ASA Ⅰ ratio between the experimental group and the control group in each included study.

### 3.3 Risk assessment of bias

**FIGURE 3:**
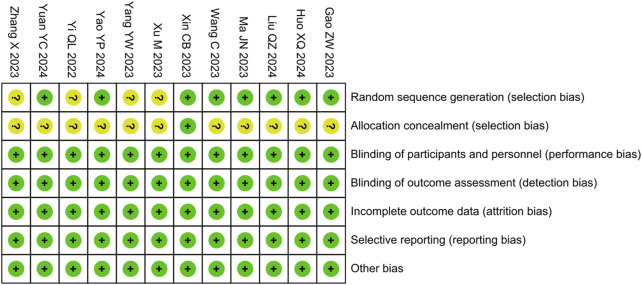
Risk of bias summary.

We assessed the risk of bias for included studies using the tool provided by the Cochrane Collaboration. Among them, four studies were evaluated as having an unclear risk of bias for random sequence generation due to not describing the randomization method, 11 studies were evaluated as having an unclear risk of bias for allocation concealment due to not describing the concealment method, whereas the rest of the domains were evaluated as having a low risk (Figure 3).

### 3.4 Meta analysis

**FIGURE 4:**
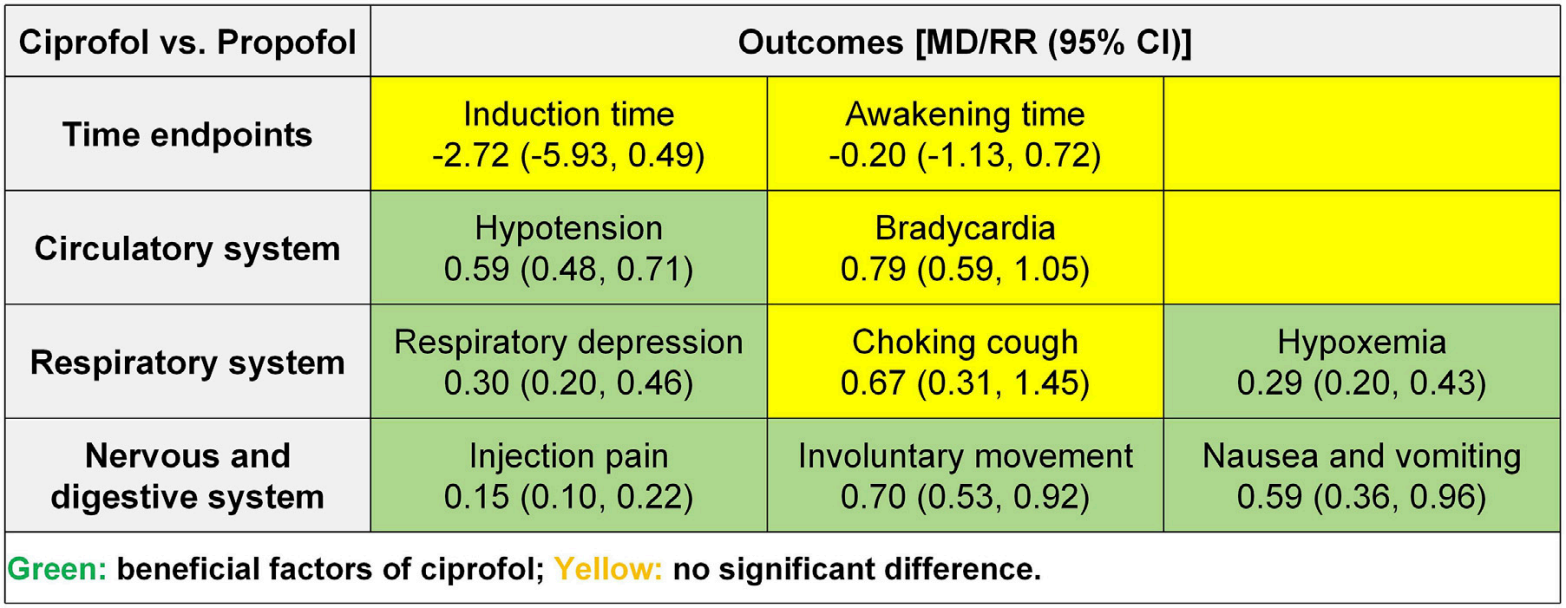
Summary of meta-analysis results.

We conducted meta-analyses on time-related outcomes, as well as cardiovascular, respiratory, neurological, and gastrointestinal outcomes, and the summary results is shown in Figure 4.

#### 3.4.1 Time-related outcomes

##### 3.4.1.1 Induction time

**FIGURE 5:**
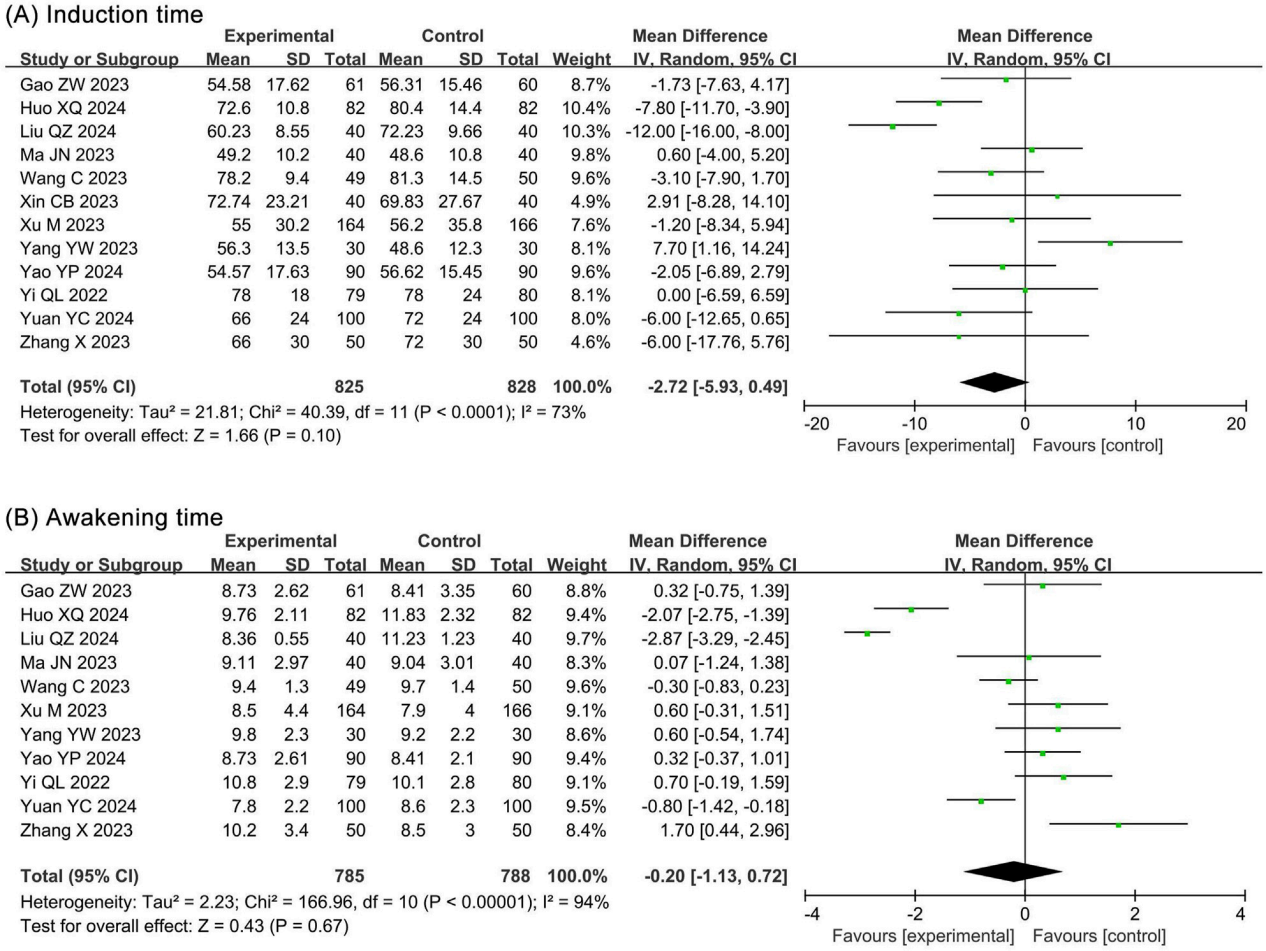
Forest plots of meta-analysis on time outcomes. **(A)** Induction time; **(B)** Awakening time.

The meta-analysis of induction times included 12 RCTs (Gao et al., 2023; Huo and Dan, 2024; Liu Q. Z. et al., 2024; Ma and Huang, 2023; Wang et al., 2023; Xin, 2023; Xu et al., 2023; Yang et al., 2023; Yao et al., 2024; Yi et al., 2022; Yuan and Chen, 2024; Zhang and Zhu, 2023) and 1,653 participants. The results showed no statistical significance in induction time (MD −2.72, 95% CI −5.93 to 0.49, *P* = 0.10, *I* ^2^ = 73%) between the ciprofol and propofol groups (Figure 5A).

##### 3.4.1.2 Awakening time

The meta-analysis of awakening times included 11 RCTs (Gao et al., 2023; Huo and Dan, 2024; Liu Q. Z. et al., 2024; Ma and Huang, 2023; Wang et al., 2023; Xu et al., 2023; Yang et al., 2023; Yao et al., 2024; Yi et al., 2022; Yuan and Chen, 2024; Zhang and Zhu, 2023) and 1,573 participants. The results showed no statistical significance in awakening time (MD −0.20, 95% CI −1.13 to 0.72, *P* = 0.67, *I* ^2^ = 94%) between the ciprofol and propofol groups (Figure 5B).

#### 3.4.2 Cardiovascular system outcomes

##### 3.4.2.1 Hypotension

**FIGURE 6:**
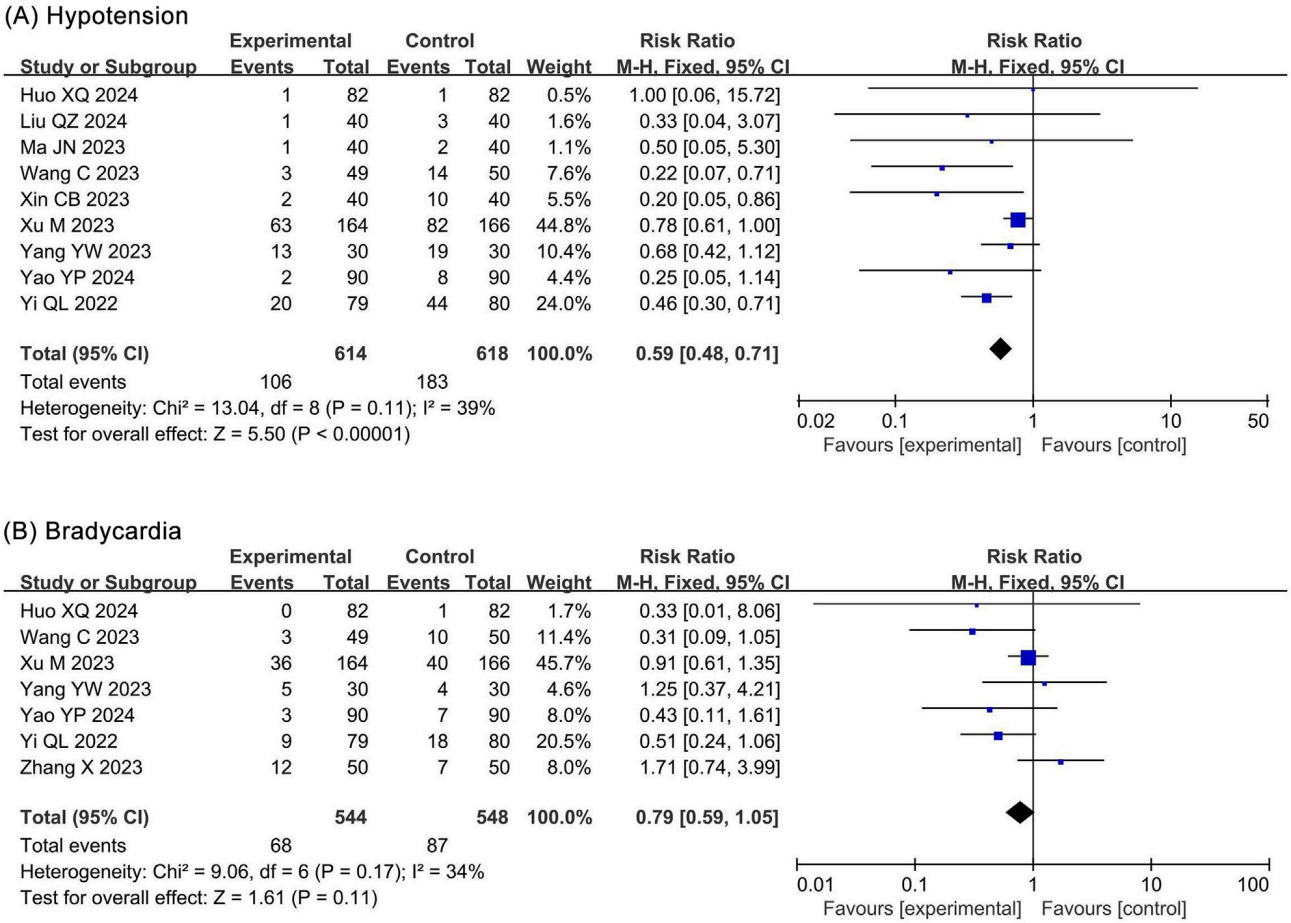
Forest plots of meta-analysis on circulatory system outcomes. **(A)** Hypotension; **(B)** Bradycardia.

The meta-analysis of hypotension included 9 RCTs (Huo and Dan, 2024; Liu Q. Z. et al., 2024; Ma and Huang, 2023; Wang et al., 2023; Xin, 2023; Xu et al., 2023; Yang et al., 2023; Yao et al., 2024; Yi et al., 2022) and 1,406 participants. The results showed a significantly lower incidence of hypotension in the ciprofol group (RR 0.59, 95% CI 0.48–0.71, *P* < 0.00001, *I* ^2^ = 39%) compared to the propofol group (Figure 6A).

##### 3.4.2.2 Bradycardia

The meta-analysis of bradycardia included 7 RCTs (Huo and Dan, 2024; Wang et al., 2023; Xu et al., 2023; Yang et al., 2023; Yao et al., 2024; Yi et al., 2022; Zhang and Zhu, 2023) and 1,092 participants. The results showed no statistical significance in bradycardia (RR 0.79, 95% CI 0.59–1.05, *P* = 0.11, *I* ^2^ = 34%) between the ciprofol and propofol groups (Figure 6B).

#### 3.4.3 Respiratory system outcomes

##### 3.4.3.1 Respiratory depression

**FIGURE 7:**
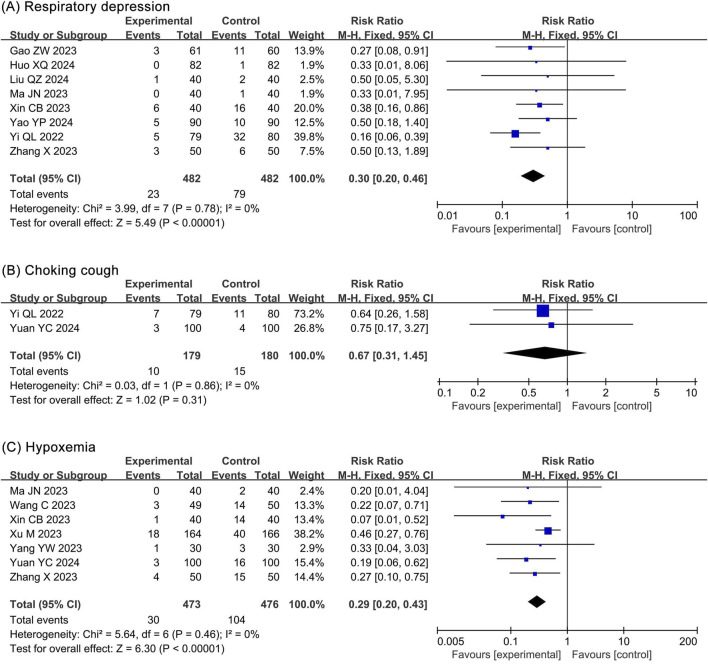
Forest plots of meta-analysis on respiratory system outcomes. **(A)** Respiratory depression; **(B)** Choking cough; **(C)** Hypoxemia.

The meta-analysis of respiratory depression included 8 RCTs (Gao et al., 2023; Huo and Dan, 2024; Liu Q. Z. et al., 2024; Ma and Huang, 2023; Xin, 2023; Yao et al., 2024; Yi et al., 2022; Zhang and Zhu, 2023) and 964 participants. The results showed a significantly lower incidence of respiratory depression (RR 0.30, 95% CI 0.20–0.46, *P* < 0.00001, *I* ^2^ = 0%) in the ciprofol group compared to the propofol group (Figure 7A).

##### 3.4.3.2 Choking cough

The meta-analysis of choking cough included 2 RCTs (Yi et al., 2022; Yuan and Chen, 2024) and 359 participants. The results showed no statistical significance in choking cough (RR 0.67, 95% CI 0.31–1.45, *P* = 0.31, *I* ^2^ = 0%) between the ciprofol and propofol groups (Figure 7B).

##### 3.4.3.3 Hyoxemia

The meta-analysis of hypoxemia included 7 RCTs (Ma and Huang, 2023; Wang et al., 2023; Xin, 2023; Xu et al., 2023; Yang et al., 2023; Yuan and Chen, 2024; Zhang and Zhu, 2023) and 949 participants. The results showed a significantly lower incidence of hypoxemia (RR 0.29, 95% CI 0.20–0.43, *P* < 0.00001, *I* ^2^ = 0%) in the ciprofol group compared with the propofol group (Figure 7C).

#### 3.4.4 Neurological and gastrointestinal system outcomes

##### 3.4.4.1 Injection pain

**FIGURE 8:**
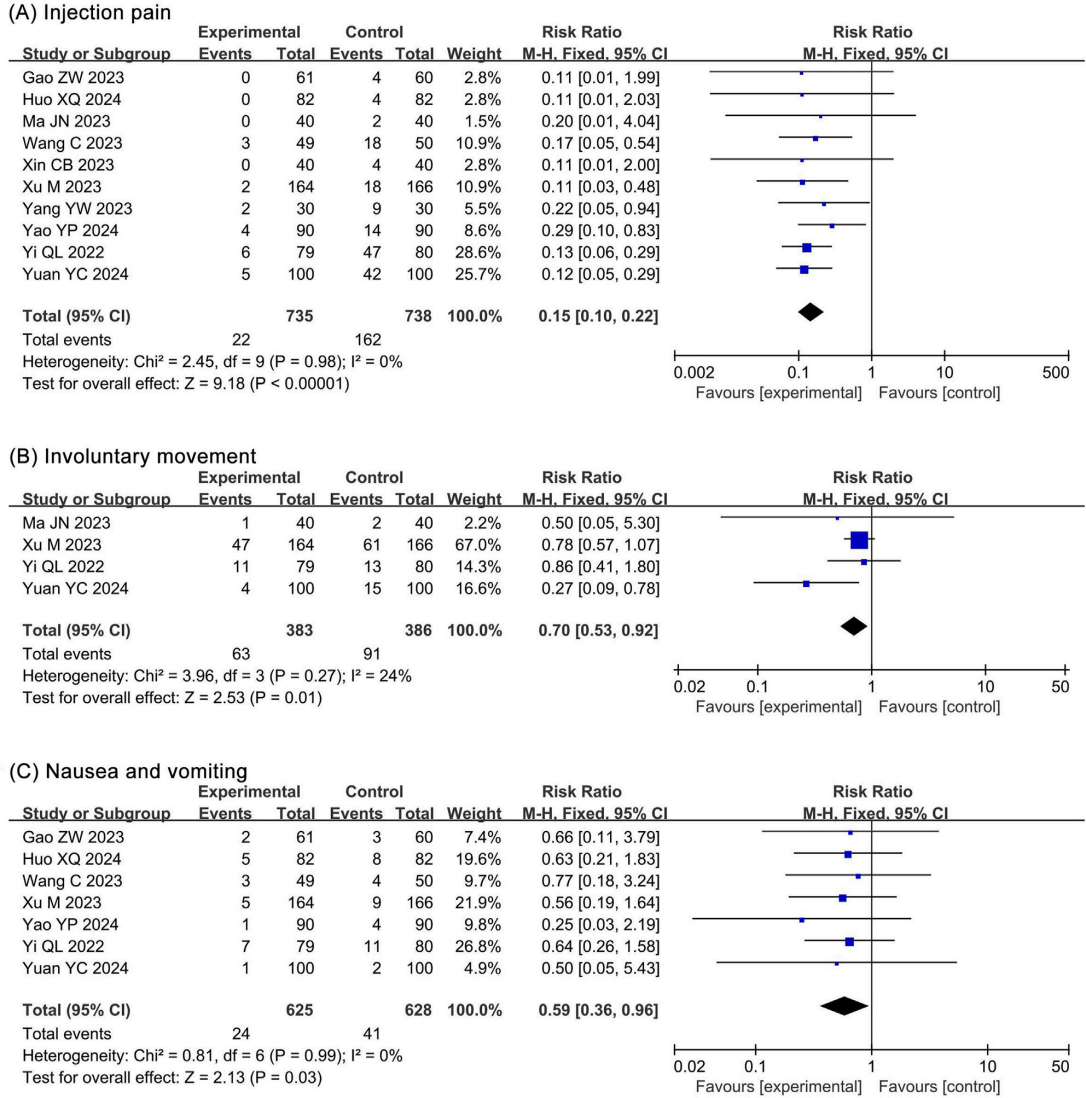
Forest plots of meta-analysis on nervous and digestive system outcomes. **(A)** Injection pain; **(B)** Involuntary movement; **(C)** Nausea and vomiting.

The meta-analysis of injection pain included 10 RCTs (Gao et al., 2023; Huo and Dan, 2024; Liu J. et al., 2024; Ma and Huang, 2023; Wang et al., 2023; Xin, 2023; Xu et al., 2023; Yang et al., 2023; Yao et al., 2024; Yi et al., 2022; Yuan and Chen, 2024) and 1,473 participants. The results showed a significantly lower incidence of injection pain (RR 0.15, 95% CI 0.10–0.22, *P* < 0.00001, *I* ^2^ = 0%) in the ciprofol group compared to the propofol group (Figure 8A).

##### 3.4.4.2 Involuntary movement

The meta-analysis of involuntary movements included 4 RCTs (Ma and Huang, 2023; Xu et al., 2023; Yi et al., 2022; Yuan and Chen, 2024) and 769 participants. The results showed a significantly lower incidence of involuntary movements (RR 0.70, 95% CI 0.53–0.92, *P* = 0.01, *I* ^2^ = 24%) in the ciprofol group compared to the propofol group (Figure 8B).

##### 3.4.4.3 Nausea and vomitting

The meta-analysis of nausea and vomiting included 7 RCTs (Gao et al., 2023; Huo and Dan, 2024; Wang et al., 2023; Xu et al., 2023; Yao et al., 2024; Yi et al., 2022; Yuan and Chen, 2024) and 1,253 participants. The results showed a significantly lower incidence of nausea and vomiting (RR 0.59, 95% CI 0.36–0.96, *P* = 0.03, *I* ^2^ = 0%) in the ciprofol group compared to the propofol group (Figure 8C).

### 3.5 Subgroup analysis

The meta-analyses showed significant heterogeneity in the results of induction time and awakening time. To explore the sources of heterogeneity, we performed subgroup analyses of induction time and awakening time based on male ratio, mean age, BMI, ASA I ratio, and the dose of ciprofol. The results of subgroup analyses that identified heterogeneous sources are shown in Table 2.

**TABLE 2:** Subgroup analyses of identified sources of heterogeneity.

Outcome	Subject	Subgroup	*I* ^2^/%	MD (95% CI)	*P* value
Induction time	BMI	BMI <24.0	5	−0.13 (−0.20, −0.07)	<0.0001
		BMI ≥24.0	48	−0.73 (−3.25, 1.79)	0.57
Awakening time	ASA I ratio	ASA I ratio ≤20%	7	0.86 (0.30, 1.43)	0.003
		ASA I ratio: 21%–30%	0	0.45 (−0.33, 1.23)	0.26
		ASA I ratio: 31%–40%	0	−2.07 (−2.75, −1.39)	<0.00001
		ASA I ratio: 51%–60%	0	−0.06 (−0.46, 0.34)	0.78

The subgroup analyses revealed that the heterogeneity of induction time was related to BMI. However, induction time was not statistically different between the two groups in the “BMI ≥24.0” subgroup (MD −0.73, 95% CI −3.25 to 1.79, *P* = 0.57, *I* ^2^ = 48%), whereas it was significantly different in the “BMI <24.0” subgroup (MD −0.13, 95% CI −0.20 to −0.07, *P* < 0.0001, *I* ^2^ = 5%). This suggests that the meta-analysis result of induction times were not robust and that ciprofol may have shortened induction times in elderly individuals with a BMI <24.0.

Moreover, the heterogeneity of awakening time was related to ASA I ratio. However, awakening time was significant in the ASA I ratio ≤20% (MD 0.86, 95% CI 0.30–1.43, *P* = 0.003, *I* ^2^ = 7%) and ASA I ratio 31%–40% (MD −2.07, 95% CI −2.75 to −1.39, *P* < 0.00001, *I* ^2^ = 0%) subgroups, whereas it was not significant in the ASA I ratio 21%–30% (MD 0.45, 95% CI −0.33 to 1.23, *P* = 0.26, *I* ^2^ = 0%) and ASA I ratio 51%–60% (MD −0.06, 95% CI −0.46 to 0.34, *P* = 0.78, *I* ^2^ = 0%) subgroups. This suggested that the meta-analysis result of awakening time was not robust.

### 3.6 Sensitivity analysis

Except for induction and awakening times, mild heterogeneity was observed in the results of hypotension and bradycardia. Therefore, we assessed the heterogeneity sources of the above outcomes using leave-one-out sensitivity analysis. The sensitivity analysis showed that the heterogeneity for bradycardia originated from the study by Zhang and Zhu (2023). However, removing the study by Zhang and Zhu (2023) resulted in a significant difference in bradycardia (RR 0.71, 95% CI 0.52–0.97, *P* = 0.03, *I* ^2^ = 13%), suggesting that the meta-analysis result of bradycardia was not robust. Furthermore, the heterogeneity of hypotension was derived from the study by Xu et al. (2023). After removing this study, there was still a significant difference in hypotension (RR 0.43, 95% CI 0.32–0.58, *P* < 0.00001, *I* ^2^ = 0%), suggesting that the results for hypotension were robust.

### 3.7 TSA

**FIGURE 9:**
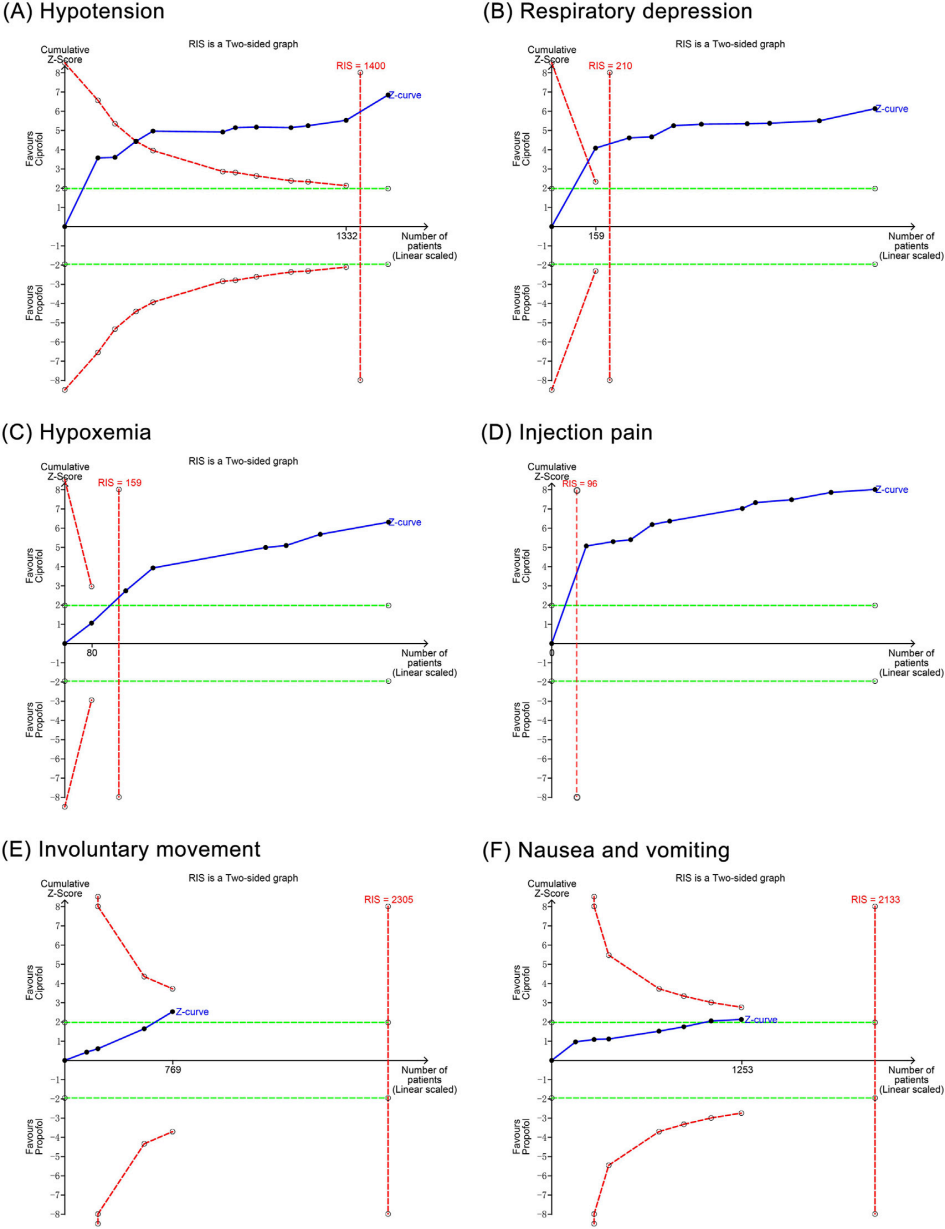
Trial sequential analysis results of positive outcomes. **(A)** Hypotension; **(B)** Respiratory depression; **(C)** Hypoxemia; **(D)** Injection pain; **(E)** Involuntary movements; **(F)** Nausea and vomiting.

The TSA showed that the cumulative Z curves for hypotension, respiratory depression, hypoxemia, and injection pain crossed the trial sequential monitoring boundary, suggesting that these outcomes observed in the current sample size were conclusive. However, the cumulative Z curves for involuntary movement as well as nausea and vomiting did not reach the trial sequential monitoring boundary, suggesting that their results observed in the current sample size are not conclusive and need to be included in new studies for further assessment (Figure 9).

### 3.8 Publication bias

**FIGURE 10:**
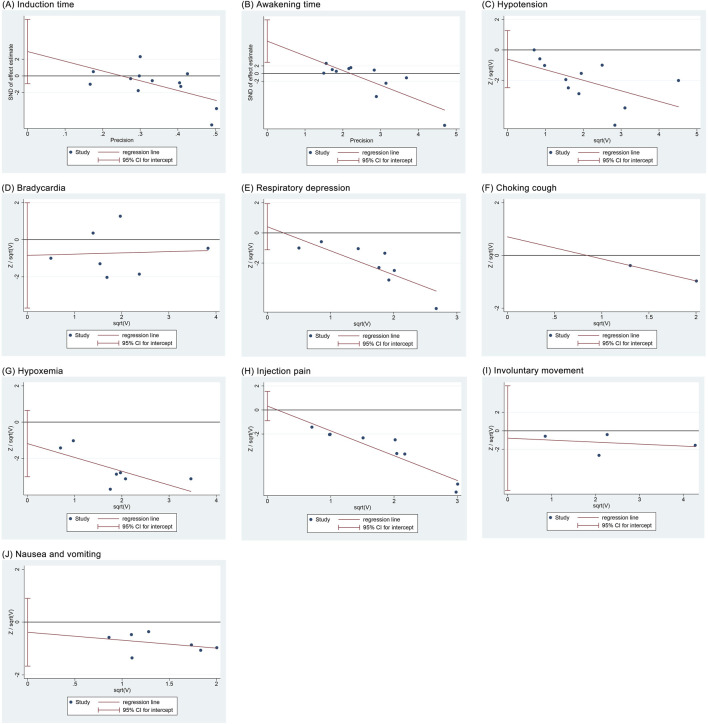
Regression analysis results of publication bias. **(A)** Induction time; **(B)** Recovery time; **(C)** Hypotension; **(D)** Bradycardia; **(E)** Respiratory depression; **(F)** Choking cough; **(G)** Hypoxemia; **(H)** Injection pain; **(I)** Involuntary movements; **(J)** Nausea and vomiting.

Regression analyses showed that induction time (*P* = 0.122), hypotension (*P* = 0.485), bradycardia (*P* = 0.478), respiratory depression (*P* = 0.548), choking cough (*P* = 1.00), hypoxemia (*P* = 0.156), injection pain (*P* = 0.555), involuntary movement (*P* = 0.607), and nausea and vomiting (*P* = 0.475) did not have significant publication bias, whereas awakening time (*P* = 0.007) had a potential publication bias (Figure 10).

### 3.9 Certainty of evidence

The GRADE showed that the qualities of evidence for hypotension, respiratory depression, hypoxemia, injection pain, and involuntary movement were moderate, those for bradycardia, choking cough, and nausea and vomiting were low, and those for induction time and awakening time were very low (Table 3).

**TABLE 3:** Certainty of evidence for outcomes.

Outcome	Risk of bias	Inconsistency	Indirectness	Imprecision	Others	Certainty of evidence
Induction time	Serious	Very serious	None	None	None	Very Low
Awakening time	Serious	Very serious	None	None	Publication bias	Very Low
Hypotension	Serious	None	None	None	None	Moderate
Bradycardia	Serious	None	None	Serious	None	Low
Respiratory depression	Serious	None	None	None	None	Moderate
Choking cough	Serious	None	None	Serious	None	Low
Hypoxemia	Serious	None	None	None	None	Moderate
Injection pain	Serious	None	None	None	None	Moderate
Involuntary movement	Serious	None	None	None	None	Moderate
Nausea and vomiting	Serious	None	None	Serious	None	Low

## 4 Discussion

### 4.1 Research background and findings

Gastrointestinal endoscopy is the mainstay of screening for gastrointestinal diseases such as esophageal, gastric, and colorectal cancers, which are prevalent among the elderly (Lambert, 2012). Although propofol is widely used for procedural sedation for gastrointestinal endoscopy, its side effects on the cardiovascular and respiratory systems remain a concern, especially in the elderly population (Guo et al., 2022). Ciprofol, a derivative of propofol, has recently been considered a potential alternative for procedural sedation in elderly population (He et al., 2024). However, this argument has not been supported by sufficient evidence due to the lack of relevant systematic reviews and meta-analyses. Therefore, we are the first to compare the effects of ciprofol and propofol on elderly patients undergoing gastrointestinal endoscopy using meta-analysis and TSA. Our findings showed that compared to propofol, ciprofol significantly reduced the incidence of hypotension, respiratory depression, hypoxemia, injection pain, involuntary movements, as well as nausea and vomiting, but had no significant effect on induction time, awakening time, bradycardia, and choking cough.

### 4.2 Effects on time related outcomes

The induction time is the duration from anesthetic drug administration to when the patient loses consciousness, reflecting the drug’s onset speed (Miller’s basics of anesthesia, 2024). In previous studies, an RCT by Ma and Huang (2023) reported that in elderly people undergoing gastrointestinal endoscopy, the induction time was almost the same in the ciprofol group and the propofol group (0.82 ± 0.17 min vs. 0.81 ± 0.18 min); and a meta-analysis by Chen et al. (2025) noted no significant difference in the induction time of ciprofol and propofol in general anesthesia for elderly people (SMD 0.11, 95% CI −0.39 to 0.61, *P* = 0.655), supporting our findings. Moreover, subgroup analyses revealed heterogeneity in induction time stemming from BMI and found that its significance was not consistent across subgroups. Specifically, the difference in induction time was significant in the BMI <24.0 subgroup (MD −0.13, 95% CI −0.20 to −0.07, *P* < 0.00001), whereas it was not significant in the BMI ≥24.0 subgroup (MD −0.73, 95% CI −3.25 to 1.79, *P* = 0.57), suggesting the meta-analysis result on induction time was not robust. This phenomenon could be related to obesity-associated alterations in cerebral hemodynamics, as increasing BMI has been shown to significantly decrease cerebral blood flow and flow velocity (Yang et al., 2011). Reduced cerebral blood flow can directly slow the delivery of ciprofol from the bloodstream to brain tissue, potentially prolonging its onset of action (Yang et al., 2011). However, ciprofol’s reduction of induction time was only 0.13 s in the BMI <24.0 subgroup, which is statistically significant but unlikely to be clinically meaningful. Therefore, although heterogeneity in induction time appears to be associated with BMI, it does not seem to influence clinical anesthetic decision-making.

The awakening time represents the duration from the end of anesthesia to the restoration of consciousness (Miller’s basics of anesthesia, 2024), reflecting the metabolic speed of the drug and the depression of the central neurological system (Miller’s basics of anesthesia, 2024). In a previous study, the RCT by Ma and Huang (2023) showed no significant difference in the awakening time between the ciprofol and propofol groups in gastrointestinal endoscopic sedation among the elderly population (9.11 ± 2.97 min vs. 9.04 ± 3.01 min), which supports our findings. However, a meta-analysis by Chen et al. (2025) found that elderly people who received general anesthesia with ciprofol had longer awakening times than those who received propofol (SMD 0.46, 95% CI 0.16–0.76); and a meta-analysis by Liu J. et al. (2024) also demonstrated that in endoscopic sedation, the awakening time was significantly longer in the ciprofol group than in the propofol group (MD 0.70, 95% CI 0.00–1.40). This contradiction was attributed to the participants, as both we and Ma and Huang (2023) focused on the elderly undergoing gastrointestinal endoscopy, whereas Liu J. et al. (2024) and Chen et al. (2025) included a wide range of patients undergoing endoscopic procedures and surgical procedures, respectively. Furthermore, subgroup analyses showed heterogeneity in awakening time stemming from ASA I ratio, suggesting that the results were not robust. Specifically, differences to awakening time in the ASA I ratio ≤20% subgroup (MD 0.86, 95% CI 0.30–1.43, *P* = 0.003) and the ASA I ratio 31%–40% subgroup (MD −2.07, 95% CI −2.75 to −1.39, P < 0.00001) were significant, whereas it was not significant in either the ASA I ratio 21%–30% subgroup (MD 0.45, 95% CI −0.33 to 1.23, *P* = 0.26) or ASA I ratio 51%–60% subgroup (MD −0.06, 95% CI −0.46 to 0.34, *P* = 0.78). However, upon reviewing the included studies, we found no clear clinical or methodological factors that could explain these discrepancies. We speculate that the heterogeneous findings might be due to the limited sample sizes within each subgroup, as only 164–589 participants were included. Consequently, the heterogeneity in awakening time related to ASA I ratio is unlikely to have significant clinical implications.

### 4.3 Effects on cardiovascular system

Our meta-analysis showed that ciprofol reduced the incidence of hypotension by 41% compared to propofol, but had no significant effect on the incidence of bradycardia. TSA suggested that the significant difference in hypotension between ciprofol and propofol was conclusive. In previous studies, Chen et al. (2025) reported a reduction in the risk of hypotension by 28% with ciprofol compared to propofol in general anesthesia (RR 0.72, 95% CI 0.58–0.94), and Liu J. et al. (2024) found that ciprofol reduced the risk of hypotension by 27% compared to propofol in endoscopic sedation (RR 0.73, 95% CI 0.58–0.92), supporting our findings. Although leave-one-out sensitivity analysis showed heterogeneity in hypotension related to the study by Xu et al. (2023), there was still a significant difference in hypotension after excluding this study (RR 0.43, 95% CI 0.32–0.58, *P* < 0.00001), indicating that the meta-analysis results were robust. The advantage of ciprofol over propofol in the risk of hypotension may be related to pharmacologic properties (Durai Samy and Taksande, 2024). Although both drugs act by activating GABA receptors, ciprofol exerts a weaker inhibitory effect on the cardiovascular system, thus avoiding drastic hemodynamic fluctuations (Petkar et al., 2024). In contrast, propofol has a multifaceted inhibitory effect on the cardiovascular system: First, propofol dilates peripheral vasculature and decreases resistance through direct action on vascular smooth muscle (Kuriyama et al., 2012; Carey et al., 2019); second, propofol decreases myocardial contractility and cardiac output through myocardial inhibition (Meng et al., 2016); and, third, propofol interferes with positive cardiovascular regulatory functions through inhibition of sympathetic nerve activity (Liu et al., 2019). These differences in pharmacological properties dictate that ciprofol possesses less cardiovascular depression and more stable hemodynamics than propofol.

Furthermore, a meta-analysis by Liu J. et al. (2024) showed no significant difference in the incidence of bradycardia between ciprofol and propofol in endoscopic sedation (RD 0.00, 95% CI −0.04 to 0.04), which supports our findings. However, a meta-analysis by Chen et al. (2025) reported a reduction in bradycardia of approximately 36% in the ciprofol group compared to the propofol group during general anesthesia surgery (RR 0.64, 95% CI 0.48–0.85), which is in contradiction to our results. This contradiction may be related to the difference in participants, as both we and Liu J. et al. (2024) included endoscopy subjects undergoing procedural sedation, whereas Chen et al. (2025) included surgical patients undergoing general anesthesia. However, the type of surgery determines the anesthetic dosage and duration, which in turn affects the incidence of bradycardia. Specifically, in endoscopic sedation, the dosage of anesthetic is relatively small and of short duration, resulting in a relatively limited effect on heart rate. Conversely, in general anesthesia procedures, the dosage of anesthetics is greater and of longer duration, which makes the patient more likely to experience bradycardia and thus sets the stage for highlighting the benefits of ciprofol in reducing bradycardia. Additionally, sensitivity analysis revealed heterogeneity in bradycardia associated with the study by Zhang and Zhu (2023), which was attributed to a low ASA I ratio. However, after excluding the Zhang and Zhu (2023) study, the difference in bradycardia incidence between the two drugs became significant (RR 0.71, 95% CI 0.52–0.97, *P* = 0.03, *I* ^2^ = 13%). This suggests that the meta-analysis results regarding bradycardia are not robust and may be influenced by the ASA I ratio. The ASA classification is a crucial measure for assessing a patient’s overall physical status and potential risk (Sankar et al., 2014), with a lower ASA I ratio indicating poorer health and a higher burden of comorbidities among participants. This implies that ciprofol may be particularly beneficial for elderly patients with fewer comorbidities, as it demonstrated a reduction in bradycardia after excluding studies with a low ASA I ratio. Patients with more comorbidities may be more sensitive to the side effects of anesthetic drugs (Hendrix and Garmon, 2025). In individuals with a lower ASA I ratio, the heart may already be under stress due to pre-existing conditions, and anesthetic agents can further impair cardiac function, increasing the likelihood of bradycardia. Therefore, the effect of ciprofol in reducing bradycardia may not be significant in patients with higher ASA classifications, especially those with cardiovascular complications. This finding provides guidance for the clinical application of ciprofol, suggesting that it may further reduce the incidence of bradycardia in ASA I patients and supports the idea of making anesthesia decisions based on ASA classification.

### 4.4 Effects on respiratory system

This study demonstrated that, compared to the propofol group, the ciprofol group reduced the incidence of respiratory depression by 70% and hypoxemia by 71%, with no significant effect on the incidence of choking cough. The TSA confirmed that the significant differences in respiratory depression and hypoxemia between ciprofol and propofol were conclusive. In a previous meta-analysis, Chen et al. (2025) reported that ciprofol reduced the risk of respiratory depression by 71% (RR 0.29, 95% CI 0.19–0.43) and hypoxemia by 62% (RR 0.38, 95% CI 0.26–0.55) in the elderly population during general anesthesia surgery compared to propofol, supporting our findings. The differences in respiratory safety between the two anesthetics may be related to the GABA receptor subtypes. GABA receptors consist of eight subunits, including α, β, γ, ρ, δ, ε, π, and θ. Except for δ, ε, π and θ, which have only one subtype, the α subunit has six subtypes and the β, γ and ρ subunits have three subtypes. The combination of different subtypes of these subunits confers GABA receptor diversity and determines its function (Chen et al., 2024; Sallard et al., 2021). When binding to GABA receptors, ciprofol selectively binds the α1, β2, and γ2 subunits to exert a sedative effect (Liao et al., 2022). This selectivity allows ciprofol to achieve sedation while attenuating inhibition of the respiratory center (Liao et al., 2022). Conversely, propofol binds a wide range of different subtypes of the α, β, and γ subunits of the GABA receptor, including subtypes that are critical for respiratory regulation (Jiang et al., 2021). For example, the β3 subtype, a key target of propofol, binds to propofol and directly inhibits respiratory-related brain nuclei function, leading to an increased risk of respiratory depression and hypoxemia (Jiang et al., 2021). This evidence provides an explanation for the respiratory benefit of ciprofol relative to propofol. Additionally, our meta-analysis reported no significant difference in the incidence of choking cough between ciprofol and propofol for the first time, filling a gap in previous meta-analyses.

### 4.5 Effects on neurological and gastrointestinal system

Our meta-analysis revealed that, compared to the propofol group, the ciprofol group significantly reduced the incidence of injection pain by 85% and involuntary movement by 30%. The TSA suggested that the significant difference between ciprofol and propofol in injection pain was conclusive, whereas the significant difference in involuntary movements needs to be validated by more studies. In previous studies, Chen et al. (2025) reported a reduction in injection pain by 87% with ciprofol compared to propofol in general anesthesia (RR 0.13, 95% CI 0.09–0.20), and Liu J. et al. (2024) noted a reduction in the incidence of injection pain with ciprofol compared to propofol by 34% in endoscopic sedation (RD −0.34, 95% CI −0.48 to −0.19), supporting our findings. The benefit of ciprofol in injection pain is closely related to its chemical structure (Usach et al., 2019). As a derivative of propofol, ciprofol replaces the methyl group of propofol with a cyclopropyl group, resulting in higher lipid solubility (Guo, 2023). This increases the solubility of propofol in the oil phase and decreases its aqueous-phase drug concentration (Doenicke et al., 1996; Sawant et al., 2021), thereby reducing irritation of the vascular intima (Doenicke et al., 1996). In contrast, propofol has a higher aqueous-phase drug concentration and thus is more likely to irritate the endothelium and local nerve endings to induce injection pain (Sim et al., 2009; Desousa, 2016).

Interestingly, the meta-analysis by Chen et al. (2025) supported the benefit of ciprofol on involuntary movements (RR 0.73, 95% CI 0.56–0.96) as we did, whereas the meta-analysis by Liu J. et al. (2024) denied this difference (RR 0.84, 95% CI 0.64–1.10). We hypothesized that this contradiction might be mediated by statistical heterogeneity and random effects models. The involuntary movements reported by us and Chen et al. (2025) were based on low heterogeneity and fixed-effects models, whereas the results of Liu J. et al. (2024) were based on random-effects models and had substantial heterogeneity. However, random effects models may amplify estimates of negative outcomes, which makes small differences difficult to detect. Additionally, while the meta-analysis supports that ciprofol reduces involuntary movements, the TSA recommends that the conclusiveness of this finding be tested by more studies of the same type.

Furthermore, our study reveals a significant reduction in nausea and vomiting by 41% in the ciprofol group compared to propofol. However, in previous meta-analyses, Chen et al. (2025) reported that ciprofol did not reduce nausea and vomiting in general anesthesia procedures (RR 0.69, 95% CI 0.43–1.11), and Liu J. et al. (2024) concluded that the incidence of nausea and vomiting was comparable between ciprofol and propofol in endoscopic sedation (RD −0.02, 95% CI −0.06 to 0.02). This contradiction may be related to the type of surgery, as Chen et al. (2025) included patients undergoing general anesthesia, and Liu J. et al. (2024) included patients undergoing a variety of endoscopic surgery including bronchoscopy, hysteroscopy, and endoscopic retrograde cholangiopancreatography. In contrast, our meta-analysis included only elderly patients undergoing gastrointestinal endoscopy. Compared to other endoscopic procedures and surgeries, gastroscopy is more prone to nausea and vomiting because it directly stimulates the pharynx and esophagus, especially the glossopharyngeal nerve in the pharynx. In this context, the stronger central inhibition of ciprofol gives it a relative advantage in reducing the incidence of nausea and vomiting. However, TSA indicated that the significant difference between ciprofol and propofol regarding nausea and vomiting lacks conclusive evidence, suggesting that this benefit needs further validation in future studies.

### 4.6 Discovery and inspiration

This meta-analysis with TSA compares the efficacy and safety of ciprofol and propofol in gastrointestinal endoscopic sedation for elderly patients, providing guidance for sedative selection in this context. Our findings indicate that in elderly patients undergoing gastrointestinal endoscopy, ciprofol significantly reduced hypotension, respiratory depression, hypoxemia, and injection pain compared to propofol, thereby benefiting the cardiovascular, respiratory, and nervous systems. Although the meta-analysis reported benefits of ciprofol regarding involuntary movements and nausea/vomiting, TSA indicated that these findings require further validation. Therefore, this study supports the conclusion that ciprofol reduces adverse cardiovascular and respiratory events, as well as injection pain, in elderly patients undergoing gastrointestinal endoscopy. However, the impact on the incidence of involuntary movements and nausea/vomiting warrants further discussion. In summary, for elderly patients undergoing gastrointestinal endoscopy, ciprofol may be a safer sedative than propofol, particularly for those with low pain thresholds and comorbid cardiovascular or respiratory disorders. Consequently, we recommend the use of ciprofol for sedation in gastrointestinal endoscopy to enhance comfort and anesthetic safety in elderly patients undergoing the procedure.

## 5 Limitations and prospects

Although our study provides evidence for the use of ciprofol in elderly patients undergoing gastrointestinal endoscopy, some limitations need to be recognized. First, four included studies did not report randomization methods and eleven studies did not report allocation concealment, which increases the risk of selectivity bias. Second, as ciprofol is currently approved and marketed only in China, the experimental centers of the included studies were all in China. This means that our findings may only apply to ethnic Chinese and need to be interpreted with caution in other races. Third, although the meta-analysis supported the benefits of ciprofol on involuntary movements as well as nausea and vomiting, the TSA suggests that these results require further validation from similar studies. Fourth, some of the included studies did not report the type and proportion of comorbidities, which prevented us from analyzing the effects of ciprofol and propofol on elderly populations with different comorbidities. Additionally, the role of ciprofol in elderly people with different comorbidities and the optimal dose range of ciprofol need to be assessed by block group randomization.

## 6 Conclusion

Our findings indicate that for elderly patients undergoing gastrointestinal endoscopy, ciprofol is associated with fewer adverse events in the cardiovascular, respiratory, and neurological systems compared to propofol. This highlights that ciprofol may be a more appropriate sedative for gastrointestinal endoscopy in the elderly. However, the optimal dose of ciprofol in gastrointestinal endoscopic sedation in the elderly remains to be explored.

## Data Availability

The original contributions presented in the study are included in the article/supplementary material, further inquiries can be directed to the corresponding authors.

## Abbreviations

AA, average age; BMI, body mass index; CI, confidence interval; CNKI, China National Knowledge Infrastructure; CSTJ, China Science and Technology Journal Database; MD, mean difference; RCT, randomized controlled trial; RR, risk ratio; SMD, standardized mean difference; TSA, trial sequential analysis; WMD, weighted mean difference.
